# GLIDERS - A web-based search engine for genome-wide linkage disequilibrium between HapMap SNPs

**DOI:** 10.1186/1471-2105-10-367

**Published:** 2009-10-31

**Authors:** Robert Lawrence, Aaron G Day-Williams, Richard Mott, John Broxholme, Lon R Cardon, Eleftheria Zeggini

**Affiliations:** 1 Wellcome Trust Centre for Human Genetics, University of Oxford, Oxford, UK; 2 Wellcome Trust Sanger Institute, Hinxton, UK; 3 Fred Hutchinson Cancer Research Center, Seattle, Washington, USA; 4 GlaxoSmithKline, King of Prussia, Pennsylvania, USA

## Abstract

**Background:**

A number of tools for the examination of linkage disequilibrium (LD) patterns between nearby alleles exist, but none are available for quickly and easily investigating LD at longer ranges (>500 kb). We have developed a web-based query tool (GLIDERS: Genome-wide LInkage DisEquilibrium Repository and Search engine) that enables the retrieval of pairwise associations with r^2 ^≥ 0.3 across the human genome for any SNP genotyped within HapMap phase 2 and 3, regardless of distance between the markers.

**Description:**

GLIDERS is an easy to use web tool that only requires the user to enter rs numbers of SNPs they want to retrieve genome-wide LD for (both nearby and long-range). The intuitive web interface handles both manual entry of SNP IDs as well as allowing users to upload files of SNP IDs. The user can limit the resulting inter SNP associations with easy to use menu options. These include MAF limit (5-45%), distance limits between SNPs (minimum and maximum), r^2 ^(0.3 to 1), HapMap population sample (CEU, YRI and JPT+CHB combined) and HapMap build/release. All resulting genome-wide inter-SNP associations are displayed on a single output page, which has a link to a downloadable tab delimited text file.

**Conclusion:**

GLIDERS is a quick and easy way to retrieve genome-wide inter-SNP associations and to explore LD patterns for any number of SNPs of interest. GLIDERS can be useful in identifying SNPs with long-range LD. This can highlight mis-mapping or other potential association signal localisation problems.

## Background

The discovery of the block structure of haplotypes has led to much research into patterns of local linkage disequilibrium (LD) in the genome [1-3]. The International HapMap Project was motivated by these discoveries to create a fine-scale catalogue of common single nucleotide polymorphisms (SNPs) in different populations to allow further investigations into LD [4,5]. The HapMap has allowed researchers to better understand patterns of LD and utilize the information in the design of genome-wide association studies (GWAS). Several tools have been developed allowing researchers to utilize the HapMap data to investigate LD, including Haploview and SNAP [6,7]. Analysis of HapMap has revealed wide-spread, and often complex, patterns of LD which has made the localization of causal variants difficult in regions identified to be associated with disease through GWAS. Research thus far has been focused on regional patterns of LD and has revealed that LD in most genomic regions decays substantially over several kilobases (Kb) to several hundreds of Kb. Long-range (> 500 Kb) LD has been less well characterized, and at present there are no known resources for researchers to investigate long-range LD. The SNAP server, for example, only allows researchers to investigate markers that are separated by a maximum of 500 Kb. Although regional patterns of LD are indeed very useful, for example in delineating intervals for the follow-up of interesting association signals, it can also be useful to examine patterns of long-range LD. There is a possibility that misplaced SNPs (or potentially epistasis) could produce very long-range cis- (intra-) and even trans- (inter-) chromosomal associations between SNPs. To address the need for a resource investigating long-range LD we have developed the Genome-wide LInkage DisEquilibrium Repository and Search engine (GLIDERS). GLIDERS is a web-based tool allowing researchers to investigate both local and long-range associations between all HapMap phase 2 and 3 SNPs.

## Construction and content

### Implementation

We computed pairwise r^2 ^and D' among all pairs of SNPs based on genotype data from HapMap2 (release 21 and 23) and HapMap3 (release 2) in three analysis panels (CEU, CHB+JPT and YRI) [4,5]. We store the physical position of each SNP, which is based on genome build 35 for HapMap2 release 21, and build 36 for HapMap2 release 23 and HapMap3 release 2. Before analysis, we performed quality control (QC), in addition to HapMap QC, to minimize genotyping artifacts. The QC analysis was based on insights gained from the Wellcome Trust Case Control Consortium (WTCCC) study [8] and excluded SNPs with ≥ 5% genotype failure rate, MAF < 5%, heterozygosity > 75%, and HWE p-value < 5.7 × 10^-7^. Additionally, HapMap3 central QC removed many SNPs investigated in HapMap2. The final number of SNPs analyzed after the above QC procedures are seen in Table 1 for each population and HapMap data-set. We based LD calculations in both HapMap2 datasets on 60 founders for the CEU and YRI populations, and 90 founders for the CHB+JPT population. We based HapMap3 LD calculations on 112 CEU, 113 YRI, and 170 CHB+JPT founders. The sample size for the LD calculations is small thereby limiting the power to detect LD. To assess the significance of the LD results GLIDERS has calculated and returns the chi-squared statistic and associated p-value (Bonferroni corrected and uncorrected) for each LD result. In addition to capturing the physical position for each SNP, we also examine its inclusion in the following commercially available genotyping arrays: Affymetrix 100K Mapping Array, Affymetrix 500K Mapping Array, Affymetrix 6.0 Array, Illumina Human-1, Illumina HumanHap300, Illumina HumanCNV370, Illumina HumanHap550, Illumina Human610, Illumina HumanHap650Y, and Illumina Human1M [9,10]. For every SNP examined GLIDERS stores information on all SNPs genome-wide (i.e. for all possible distances along a chromosome as well as across chromosomes) with an r^2 ^≥ 0.3. GLIDERS does not handle SNP aliasing created by dbSNP updating. The data are stored as text files in a tree-based directory structure for fast query performance. Our analysis shows a nearly linear-time query performance with the number of query SNPs. The parameters that affect query performance are the distance and r^^2^parameters. The data are accessed and queried by a Perl CGI script.

**Table 1:** HapMap SNPs analyzed for genome-wide LD post-QC.

**HapMap Data-set**	**CEU**	**YRI**	**CHB+JPT**
HapMap2 r21	1937009	2130827	1733922
HapMap2 r23	2021959	2202571	1824262
HapMap3 r2	1181659	1261371	1086818

This table shows the number of SNPs that passed QC and were analyzed for genome-wide LD patterns for each population in the three HapMap datasets.

### Web Server

**Figure 1:**
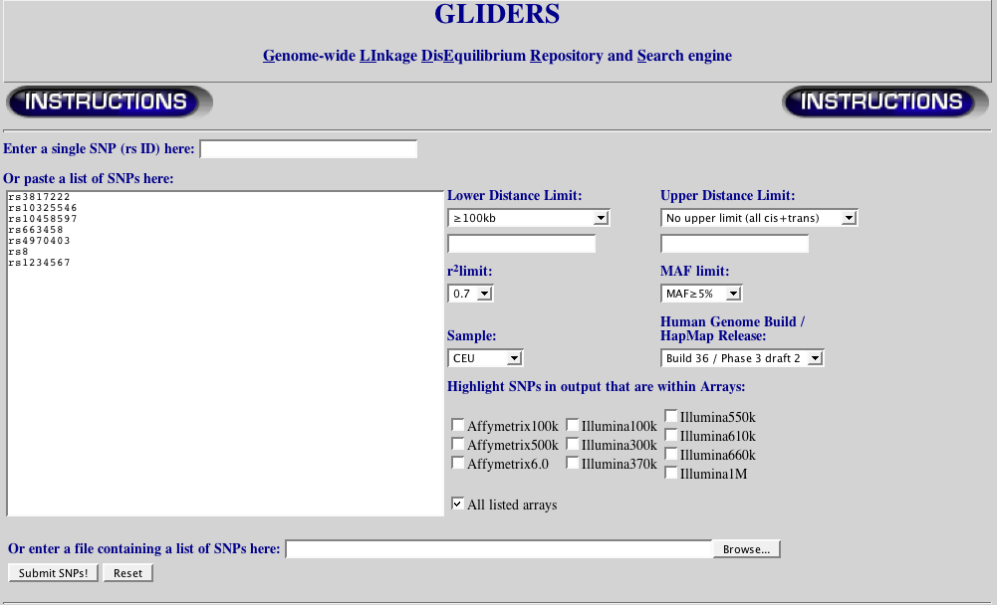
**GLIDERS home page**. The top of the home page provides a link to detailed instructions about how to use the application. Below the instructions link are the search configuration controls. The user can select the HapMap population and HapMap release to query, and the user can restrict the results based on distance, MAF, and r^2 ^values. The user can also select to be informed of the inclusion of the resulting SNP(s) on several commercial genotyping arrays.

**Figure 2:**
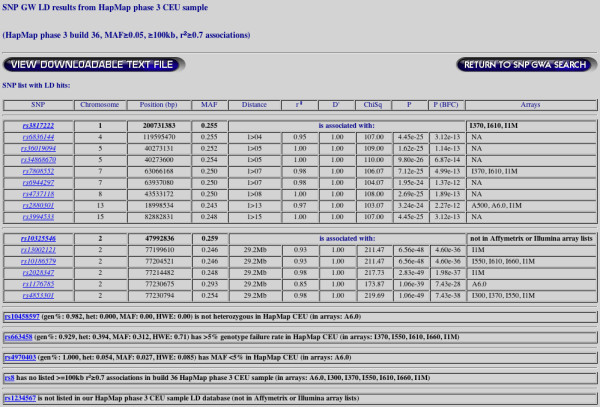
**GLIDERS results page**. The top of the page displays the HapMap phase, build, and population queried, as well as any user specified query restrictions. Below the query information are the results, with the results for each query SNP in its own table. The first SNP in each table is the query SNP, followed by all the SNPs in the genome that meet the search criteria. The information displayed for each SNP includes the chromosome, base pair position, MAF, distance from query SNP, r^2 ^with query SNP, D' with query SNP, chi-squared statistic and p-value (Bonferroni corrected and uncorrected), and information about its membership on selected commercial arrays. Additional information on each SNP can be obtained by clicking the SNP name which takes the user to the dbSNP record for that SNP. The results page also informs users if any query SNP failed the QC criteria, is not a SNP in HapMap, or whether no SNPs met the specified search criteria. The user is also provided with a link at the top and bottom of the page to download a tab-delimited text file of the results.

The GLIDERS search engine is publicly available at . Detailed documentation can be accessed by a link at the top of the page and includes examples of how to use the application. Users can select which HapMap version and release, and which study population they want to investigate from drop-down lists as seen in Figure 1 (default selections are HapMap3 release 2 and CEU). The user can then enter query SNP(s) by manually entering rsIDs or by uploading a text file of rsIDs. GLIDERS allows users to further restrict their analysis by filtering the results on a MAF cut-off for all returned SNPs, a minimum distance between SNPs, a maximum distance between SNPs, and a minimum r^2 ^value between SNPs. The user is then presented with a table of results, as seen in Figure 2, for each of the query SNPs entered. For each query SNP entered, GLIDERS returns all the SNPs that satisfy the user-defined filters and displays their chromosome, position in base-pairs, MAF, distance from the query SNP in base-pairs, r^2 ^with query SNP, D' with the query SNP, and all the commercially available chips that the SNP is included in. Further information is available by clicking the rsID, which takes the user to the dbSNP record for that SNP [11]. In addition to the web-based table, users can download a text file of the results by clicking a button at the top or bottom of the results page. If any of the query SNPs were not analyzed because they failed the QC analysis detailed above, the user is informed and told which of the QC filters the SNP did not pass. Users are also informed if a query SNP cannot be located in the HapMap data.

## Utility and Discussion

GLIDERS is an efficient and easy tool allowing researchers to find genome-wide LD between all SNPs investigated in HapMap phase 2 and 3. GLIDERS is not the only tool for interrogating LD in the HapMap data, but it has certain features that set it apart. Firstly, GLIDERS does not require users to download and install software on their local machine, like the popular Haploview program requires[6]. Additionally, GLIDERS has pre-calculated and compiled all the LD information therefore taking the computational burden off the individual researcher. It would also be possible for researchers to perform the same analysis using Haploview by downloading all the phased HapMap genotypes, setting the maximum distance parameter to 0, and exporting the LD values. Then the researcher would have to parse all of that output. Of the available LD resources, SNAP [7] is the tool that most closely resembles GLIDERS. SNAP is a very good utility but is limited to investigating LD between markers that are a maximum of 500 Kb apart, whereas GLIDERS has an unlimited distance search space. However, because of the greatly expanded distance search space, GLIDERS has an r^2 ^lower bound of 0.3, whereas SNAP has an unlimited r^2 ^search space for SNP associations. Therefore, GLIDERS should be viewed as a complementary tool to SNAP. GLIDERS provides a quick list of potential proxy SNPs and an indication of the extent of LD between the potential proxies and the query SNP(s). GLIDERS also provides insight into potential SNP mapping errors or more interesting biological processes by revealing strong associations between SNPs on different chromosomes. The number of HapMap phase 3 SNPs demonstrating at least one trans-chromosomal association with r2 ≥ 0.3 in the three panel populations are: 17,562 in CEU, 27,064 in YRI, and 1,497 in JPT+CHB. These associations either reveal mapping errors, interesting biology, or that these populations are still young populations that have not had enough time to degrade the LD amongst unlinked loci. Our analysis has also revealed potential data quality issues between HapMap2 and HapMap3, since many of the long-range associations we discovered in the HapMap2 data disappear in HapMap3. This, we believe, is likely to be due to the increased sample size in phase 3 and the removal of poorly performing phase 2 SNPs from phase 3. Therefore we recommend querying your SNPs against HapMap3, with the trade off that there are fewer SNPs analyzed in HapMap3 because of the more stringent QC analysis.

## Conclusion

With the large number of GWAS being carried out and the large number of SNPs showing disease association, it is important to be able to check quickly and easily for distant SNP proxies which might affect disease gene localization. GLIDERS is an ideal utility for this as it contains all genome-wide inter-SNP associations between HapMap markers from the three main population samples calculated using both phase 2 and phase 3 genotypes. GLIDERS is an easy to use inter-SNP association web database tool that can be used by any researcher with internet access.

## Availability and requirements

The GLIDERS search engine is publicly available at . There are no restrictions to the use of GLIDERS by academic or commercial entities.

## List of abbreviations

GWAS: (genome-wide association study); LD: (linkage disequilibrium); MAF: (minor allele frequency); SNP: (single nucleotide polymorphism); GLIDERS: (Genome-wide LInkage DisEquilibrium Repository and Search engine); CEU: (CEPH (Utah residents with ancestry from northern and western Europe)); YRI: (Yoruba in Ibadan, Nigeria); JPT: (Japanese in Tokyo, Japan); CHB: (Han Chinese in Beijing, China); CGI: (Common Gateway Interface); SNAP: (SNP Annotation and Proxy Search); rsID: (reference SNP identification); QC: (quality control); Kb: (kilobase).

## Authors' contributions

RL carried out LD calculations, developed the database and drafted the manuscript. ADW drafted the manuscript and contributed to the web server set-up. RM provided software for LD calculation. JB contributed to website design. LC contributed to database design and supervised the project. EZ supervised the project and drafted the manuscript. All authors have read and approved the manuscript.
